# THAT’S ONE WAY TO DO IT: THE EXPERIENCE OF SUCCESSFULLY PURSUING AND IMPLEMENTING THE AFU DESIGNATION

**DOI:** 10.1093/geroni/igae098.1756

**Published:** 2024-12-31

**Authors:** Katarina Friberg-Felsted

**Affiliations:** University of Utah, Salt Lake City, Utah, United States

## Abstract

Gerontology education and research has been present at the University of Utah (UofU) for 51 years. This has led to multiple programs, staff, faculty, and alumni desiring to improve age-friendly environments, advocate for lifelong learning, and choosing to pursue the Age-friendly University (AFU) designation. This presentation will describe the experience of successfully pursuing and implementing AFU at the UofU. A matrix of age-friendly initiatives across campus, representing each of the 10 Age-Friendly University Principles, was developed and used to garner support from vital entities such as the Professors Emeriti Club, the Dean of the College of Nursing, and the Center on Aging. These partners helped facilitate presentations to the Senior Vice President and the university’s Academic Council of Deans, leading to endorsement from the UofU President. Following receipt of AFU designation, AFU representatives worked with the university’s Marketing and Communications team on a campus wide press release. Strategic planning for improving age-friendliness across campus is ongoing, focusing on three main initiatives: 1) piloting a baseline needs assessment of age inclusivity, 2) creating an Age-friendly University task force, and 3) exploring a collaboration with the Associate Chief of Age-friendly Health Systems to standardize an Age-friendly model for our academic health center.

